# Diabetes Leads to Alterations in Normal Metabolic Transitions of Pregnancy as Revealed by Time-Course Metabolomics

**DOI:** 10.3390/metabo10090350

**Published:** 2020-08-27

**Authors:** Jacquelyn M. Walejko, Anushka Chelliah, Maureen Keller-Wood, Clive Wasserfall, Mark Atkinson, Anthony Gregg, Arthur S. Edison

**Affiliations:** 1 Department of Biochemistry & Molecular Biology, University of Florida, Gainesville, FL 32610, USA; 2 Department of Obstetrics, Gynecology, and Reproductive Sciences, University of Texas Health Science Center at Houston, UT Health, Houston, TX 77030, USA; anushka.chelliah@uth.tmc.edu; 3 Department of Pharmacodynamics, University of Florida, Gainesville, FL 32610, USA; kellerwd@cop.ufl.edu; 4 Department of Pathology, Immunology, and Laboratory Medicine, Diabetes Institute, University of Florida, Gainesville, FL 32610, USA; wasserfa@pathology.ufl.edu (C.W.); atkinson@pathology.ufl.edu (M.A.); 5 Department of Obstetrics and Gynecology, Baylor University, Dallas, TX 75246, USA; Anthony.Gregg@BSWHealth.org; 6 Departments of Genetics and Biochemistry & Molecular Biology, Complex Carbohydrate Research Center, University of Georgia, Athens, GA 30602, USA

**Keywords:** diabetes, pregnancy, metabolomics

## Abstract

Women with diabetes during pregnancy are at increased risk of poor maternal and neonatal outcomes. Despite this, the effects of pre-gestational (PGDM) or gestational diabetes (GDM) on metabolism during pregnancy are not well understood. In this study, we utilized metabolomics to identify serum metabolic changes in women with and without diabetes during pregnancy and the cord blood at birth. We observed elevations in tricarboxylic acid (TCA) cycle intermediates, carbohydrates, ketones, and lipids, and a decrease in amino acids across gestation in all individuals. In early gestation, PGDM had elevations in branched-chain amino acids and sugars compared to controls, whereas GDM had increased lipids and decreased amino acids during pregnancy. In both GDM and PGDM, carbohydrate and amino acid pathways were altered, but in PGDM, hemoglobin A1c and isoleucine were significantly increased compared to GDM. Cord blood from GDM and PGDM newborns had similar increases in carbohydrates and choline metabolism compared to controls, and these alterations were not maternal in origin. Our results revealed that PGDM and GDM have distinct metabolic changes during pregnancy. A better understanding of diabetic metabolism during pregnancy can assist in improved management and development of therapeutics and help mitigate poor outcomes in both the mother and newborn.

## 1. Introduction

During pregnancy, adaptations in endocrine and cardiovascular physiology contribute to a dynamic metabolic state for the mother. In addition to an increase in basal metabolic rate, maternal lipids rise in the serum throughout healthy gestation [1,2]. These lipids serve as an energy source for the mother, resulting in increased glucose and amino acid delivery to the developing fetus. The emergence of metabolomics studies has confirmed these metabolic alterations in the mother throughout gestation, providing additional evidence of decreasing amino acids and increasing tricarboxylic acid (TCA) cycle intermediates during healthy pregnancy [3,4]. Furthermore, maternal cortisol concentrations rise throughout gestation and, along with insulin resistance, result in increased circulating glucose, which ultimately supplies the developing fetus [5]. Therefore, underlying chronic diseases such as type 1 diabetes (T1D) or type 2 diabetes (T2D) are often worsened during pregnancy, leading to greater challenges in achieving glycemic control. In addition, pregnancy can lead to the development of diabetes, termed gestational diabetes mellitus (GDM), which often resolves following delivery; women who develop GDM are at a 60% increased risk of developing T2D later in life [6]. The current standard for treatment of hyperglycemia in women with pre-gestational diabetes (PGDM) involves intensive insulin therapy; occasionally, oral hypoglycemic agents, a change of diet, and close monitoring of blood glucose are recommended in women with T1D, T2D, or GDM. However, the effect of excess glucose on fetal growth is not completely understood. Both gestational and pre-gestational diabetes account for significant maternal and perinatal morbidity and mortality [7,8,9]. Hence, diabetes results in prolonged hospital stays for the mother, complicated delivery, damage to vital organs, infection, premature delivery, prolonged neonatal intensive care unit stays, increased risks of birth defects, and lifelong adverse health outcomes [10]. Pre-existing diabetes (T1D or T2D) complicates 1–2 percent of all pregnancies [11], with the number of pregnancies complicated by T2D projected to rise in the coming years [12], while GDM affects up to 10 percent of all pregnancies in the United States [13]. Therefore, as the prevalence of diabetes during pregnancy rises, the role of maternal hyperglycemia and its effect on metabolism throughout gestation, as well as the placenta and fetus, must be determined.

Metabolomics studies have already been utilized to study metabolic changes in uncomplicated pregnancies using maternal serum and urine [3,4,14]. In addition, metabolomics has been utilized to determine metabolic pathways important in T2D development in women that had GDM as well as metabolites in cord blood associated with GDM development [15,16,17]. However, to date, there are no published studies regarding the effects of diabetes, either PGDM or GDM, on global metabolism across gestation and the impact of these changes in maternal metabolism on the placental and fetal metabolome. Therefore, the objective of this study was to utilize metabolomics to identify metabolic pathways altered in women with diabetes throughout pregnancy, and to determine whether these metabolic alterations are also present in the cord blood at term. We hypothesize that the normal metabolic trajectory in pregnancy will be disrupted in women with either PGDM or GDM, but that the disruption will differ between these two etiologies of diabetes.

## 2. Results

### 2.1. Metabolomic Analysis Reveals Maternal Metabolic Alterations in Serum across Gestation in Women with and without Diabetes

**Figure 1:**
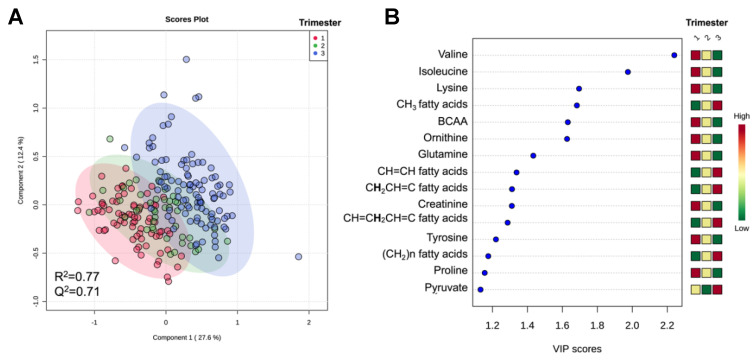
Partial least squares discriminant analysis (PLS-DA) reveals separation of maternal serum specimens from women across gestation. (**A**) The PLS-DA scores plot reveals separation of first- (*n* = 75; red), second- (*n* = 42; green), and third-trimester (*n* = 104; blue) serum specimens. (**B**) Variable importance of projection (VIP) plot for the first 15 metabolites that contribute to separation in PLS-DA component 1. A higher VIP score indicates a greater contribution of that metabolite to the separation of the groups.

**Figure 2:**
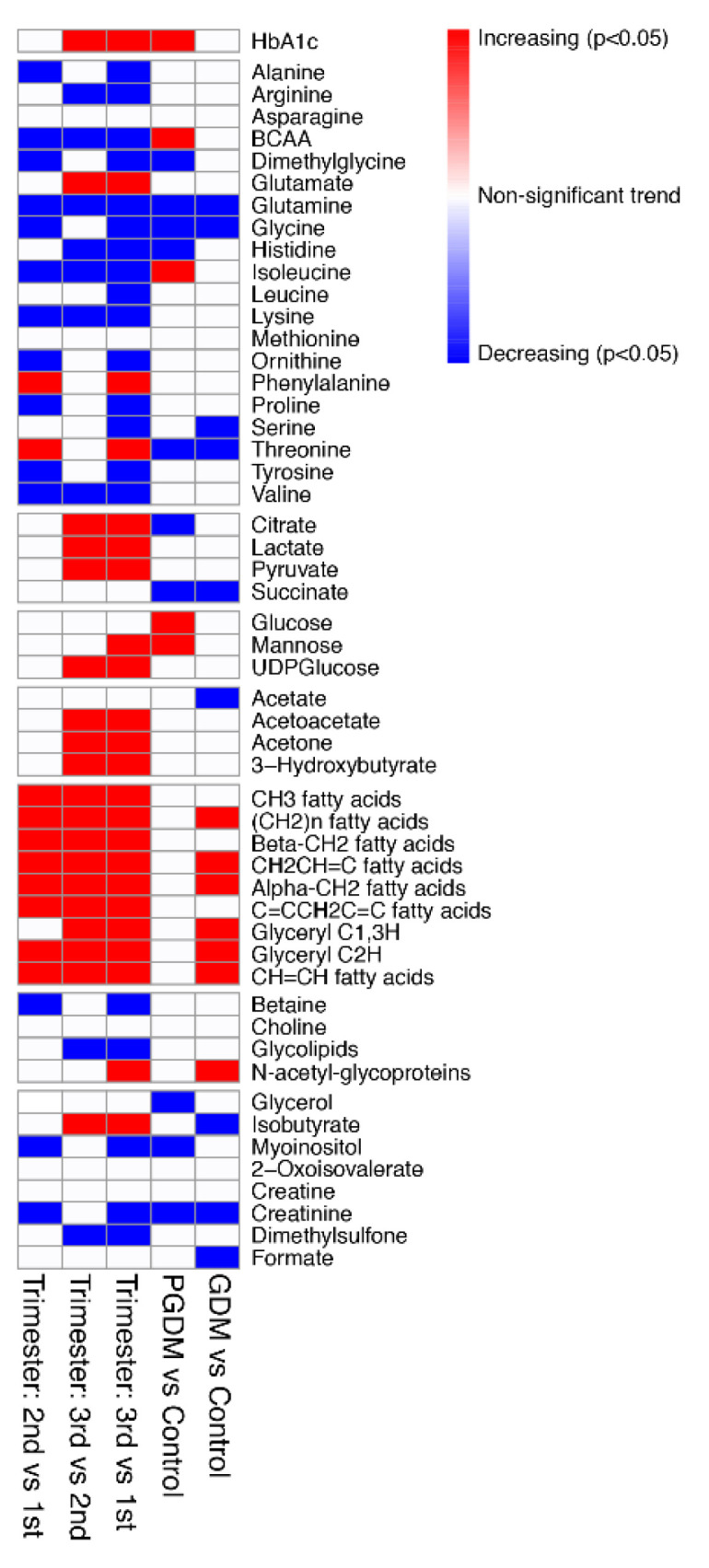
Heatmap of trimester, PGDM, and GDM metabolic alterations during pregnancy. The first three columns represent results of a post-hoc analysis of metabolites that differed between first, second, and third trimesters in all women during gestation, regardless of diabetes diagnosis. The last two columns represent metabolites altered between women with PGDM compared to controls or GDM compared to controls across all trimesters. Metabolites with a significant *p*-value (*p* < 0.05) are represented by red if they are increased and blue if they are decreased. If no significant change exists, the metabolites are shown in white. Many metabolites significantly change during each trimester in all subjects (Table 2). Significant metabolites based on PGDM compared to controls or GDM compared to controls are listed in Table 3.

We identified 52 metabolites in maternal serum during pregnancy using ^1^H-NMR (Supplementary Table S1). This includes nine reproducible and quantifiable resonances that correspond to different saturated and unsaturated lipid spin systems (Supplementary Table S1). For simplicity, we refer to these as distinct lipid features, but it should be noted that we have neither identified the lipids nor verified whether individual or multiple lipids species contribute to these resonances. A partial least squares discriminant analysis (PLS-DA) of all samples revealed separation of metabolites based on trimester, regardless of diabetes diagnosis (Figure 1A). The variable importance project plot (VIP) revealed increases in lipids and pyruvate across gestation, whereas amino acids and creatinine were decreased (Figure 1B). It should be noted that results of the PLS-DA model were not used to determine significant metabolites. A linear mixed-effects model of samples from all trimesters (corrected for maternal BMI) revealed significant increases in TCA cycle intermediates, carbohydrate, ketones, and lipids, while amino acids were significantly diminished over the course of gestation (Figure 2 and Table 1). Furthermore, hemoglobin A1c (HbA1c) levels significantly increased over the course of gestation in all subjects (including controls), consistent with elevated circulating glucose levels as gestation progresses (Figure 2 and Table 1). All results from the linear mixed-effects model of samples from all trimesters are listed in Supplementary Table S2.

### 2.2. Metabolomic Analysis Reveals Maternal Metabolic Alterations across Gestation in Women with PGDM

**Figure 3:**
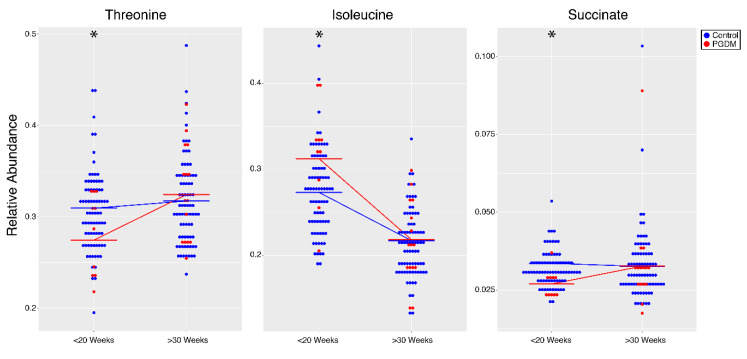
Dot plots of metabolites with interaction effects for PGDM (*n* = 10, <20 weeks; *n* = 15, >30 weeks; **red**) compared to non-diabetic controls (*n* = 78, <20 weeks; *n* = 77, >30 weeks; **blue**) during pregnancy. Significant interactions (*p* < 0.05) between PGDM and controls are represented by stars. Results for all metabolites that had significant interaction effects are listed in Table 3.

We next sought to determine which metabolites were altered in women with pre-gestational diabetes (PGDM) during pregnancy compared to non-diabetic controls. A linear mixed-effects model revealed significant elevations in HbA1c, BCAAs (including isoleucine), and sugars (glucose and mannose) in PGDM compared to controls across both time points. In addition, four amino acids (threonine, histidine, glycine, and glutamine) and two TCA cycle intermediates (succinate and citrate), as well as creatinine, myoinositol, and dimethylglycine, were significantly diminished in PGDM across both time points (Figure 2 and Table 2). HbA1c, threonine, isoleucine, acetate, acetone, and succinate had significant interaction effects between early and late gestation in PGDM compared to controls (Figure 3 and Table 3). Metabolites associated with ketone body production, including acetate and acetone, were significantly elevated in late gestation (>30 weeks) in PGDM, suggesting impaired glucose control close to delivery (Figure 3; Table 3). Isoleucine was significantly elevated in PGDM in the first 20 weeks of pregnancy, whereas threonine and succinate were significantly diminished in PGDM at this time point (Figure 3 and Table 3). Finally, pathway analysis using metabolites significantly altered in PGDM compared to controls revealed alterations in five pathways across gestation, including those involved in carbohydrate and amino acid metabolism (Table 4). All results from the linear mixed-effects model of samples from PGDM and controls during pregnancy are listed in Supplementary Table S3.

### 2.3. Metabolomic Analysis Reveals Maternal Metabolic Alterations Across Gestation in Women with GDM

**Figure 4:**
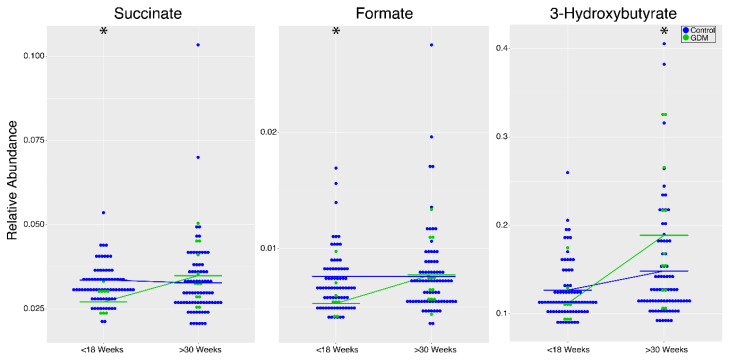
Dot plots of metabolites with interaction effects for GDM (*n* = 8, <18 weeks; *n* = 12, >30 weeks; green) compared to non-diabetic controls (*n* = 74, <18 weeks; *n* = 77, >30 weeks; blue) during pregnancy. Significant interactions (*p* < 0.05) between GDM and controls are represented by stars. Results for all metabolites that displayed significant interaction effects are listed in Table 4.

In women with gestational diabetes (GDM), six lipids, as well as *N*-acetyl-glycoproteins, were significantly elevated during pregnancy, whereas four amino acids (glycine, serine, threonine, and glutamine) as well as succinate, formate, isobutyrate, acetate, and creatinine were significantly diminished compared to non-diabetic controls across both time points (Figure 2 and Table 2). Ketones (acetoacetate, 3-hydroxybutyrate, acetate), formate, glucose, succinate, and glutamine had significant gestational age and diabetes interaction effects in GDM compared to controls (Figure 4 and Table 3). Acetoacetate and 3-hydroxybutryate, both ketone bodies, as well as glucose, were elevated in late gestation (>30 weeks) in GDM (Figure 4 and Table 3). Succinate and formate were significantly elevated in early gestation (<18 weeks) in GDM (Figure 4 and Table 3). Finally, pathway analysis using metabolites significantly altered in GDM compared to controls revealed alterations in five pathways during pregnancy, including those involved in carbohydrate, amino acid, and lipid metabolism (Table 4). All results from the linear mixed-effects model of samples from GDM and non-diabetic controls during pregnancy are listed in Supplementary Table S4.

Additionally, we wanted to identify metabolites that differed between GDM and PGDM during pregnancy. A linear mixed-effects model revealed that HbA1c and isoleucine were significantly diminished during pregnancy in GDM compared to PGDM. All results from this linear mixed-effects model are listed in Supplementary Table S5.

### 2.4. Metabolomic Analysis Reveals Maternal Metabolic Alterations in Women with PGDM or GDM Immediately Following Delivery

In PGDM, HbA1c, sugars (mannose and glucose), ketone bodies (acetoacetate and 3-hydroxybutyrate), and lipids were significantly elevated in the immediate post-partum period (within two days of delivery) compared to controls (Table 5). Amino acids including histidine, tyrosine, asparagine, glutamine, ornithine, and methionine, as well as creatinine were significantly diminished in the post-partum period in PGDM (Table 5). All results from the linear regression model of post-partum samples from PGDM and controls are listed in Supplementary Table S6. Pathway analysis using significantly altered metabolites revealed seven significant pathways during the post-partum period, including two pathways that were also altered across gestation; galactose metabolism and alanine/aspartate/glutamate metabolism (Table 6). However, five pathways were unique to the post-partum period in PGDM, including three involved in amino acid metabolism, one in carbohydrate metabolism, and one in lipid metabolism (Table 6). We next sought to determine whether women with GDM during pregnancy had altered metabolic profiles following delivery compared to non-diabetic controls. A linear regression model revealed significant elevations in mannose and HbA1c in the immediate post-partum period in GDM (Table 5). All results from the linear regression model of post-partum samples from GDM and controls are listed in Supplementary Table S7. Additionally, we wanted to determine whether women with GDM had different altered metabolic profiles following delivery compared to women with PGDM (Supplementary Table S8). A linear regression model revealed significant elevations in five amino acids (asparagine, leucine, lysine, ornithine, and tyrosine) in GDM compared to PGDM. In addition, HbA1c and *N*-acetyl glycoproteins were significantly diminished in the post-partum period in GDM. All results from the linear regression model of post-partum samples from GDM and PGDM are listed in Supplementary Table S8.

### 2.5. Cord Blood from Newborns of Women with PGDM or GDM Reveals Similar Metabolic Patterns

A total of 52 metabolites were identified in serum from the umbilical cord collected immediately following birth using NMR spectroscopy (Supplementary Table S1). Mannose, glucose, and dimethylamine were significantly elevated in PGDM cord blood compared to controls (Table 7). In addition, mannose, dimethylglycine, and betaine were significantly elevated, while glycine was significantly diminished in GDM cord blood compared to controls (Table 7). Finally, PGDM cord blood had elevated isobutyrate, alanine, and lactate compared to GDM cord blood (Supplementary Table S9). All results are listed in Supplementary Table S9.

## 3. Discussion

### 3.1. Amino Acids, TCA Cycle Intermediates, Carbohydrates, and Lipids Are Altered during Pregnancy Regardless of Diabetes Diagnosis

Metabolomic analysis of maternal serum throughout pregnancy revealed significant elevations in TCA cycle intermediates, carbohydrates and lipids. However, with the exception of glutamate and phenylalanine, amino acids decreased throughout gestation, suggesting increased utilization by the placenta and fetus. This is consistent with previous evidence of a net flux of most amino acids across the placenta to the fetus, with the exception of aspartate and glutamate [18,19]. While we did not identify aspartate in our study, glutamate was significantly elevated across gestation in all subjects. Glutamate has been shown to be produced by the fetal sheep liver in late gestation, with a net flux from the fetus to the ovine placenta [19,20]. In addition, in vitro perfusions of the human placenta revealed rapid clearance of glutamate and aspartate from fetal circulation [21], and metabolomics studies in maternal serum revealed significant elevations of glutamate during the third trimester of pregnancy [3]. Phenylalanine, an essential amino acid, has been shown to readily cross the placenta, with elevated concentrations in the fetal circulation relative to the maternal [18,19]. Therefore, the cause of elevated phenylalanine across gestation in our population is not completely clear. Future studies are needed to determine whether this elevation is due to alterations in maternal physiology during pregnancy or changes in placental transport and metabolism.

In contrast to amino acids, lipids were elevated during pregnancy in maternal serum. These results are consistent with a previous study in a non-longitudinal cohort of healthy women showing that circulating maternal lipids increase across gestation [4], which has been attributed to increased lipolysis in the mother as a result of insulin resistance [22]. In addition to lipids, our study found elevations in ketones across gestation. This supports evidence that maternal lipolysis is elevated during fasting conditions, leading to increased ketogenesis in mid-to-late gestation [23,24]. However, metabolites involved in choline metabolism, including glycerophosphocholine (GPC), phosphocholine (PC), betaine, and myo-inositol, were diminished across gestation in all mothers. Choline levels are high in the fetus [25] and along with other forms of phosphocholine, are important in brain and neural tube development [26]. Choline has been previously shown to increase in maternal serum across gestation [4,27], while phosphocholines are significantly diminished [3]. While choline was not significantly altered across gestation in maternal serum from our population, decreases in storage forms of choline (i.e., GPC and PC) in the maternal circulation, as well as betaine, a metabolite formed during choline metabolism, and myo-inositol, an important component in lipid membrane structure, suggest increased placental and fetal uptake.

TCA cycle intermediates, including pyruvate, lactate, and citrate, were elevated during pregnancy, consistent with previous metabolomic studies of maternal serum from healthy women throughout pregnancy [3,4]. These elevations may be from an increase in the basal metabolic rate of the mother to further supply the fetus with important substrates needed for development [28]. Still, the importance of these TCA cycle intermediates in the placenta and fetus are unknown. In addition, we did not observe a rise in glucose over the course of gestation in our population, even when controlling for diabetes. This is likely due to the fact that samples were collected during routine blood draws in the clinic and, therefore, included a mixed population of fasted and non-fasted individuals. However, in all samples we did see a significant increase in hemoglobin A1c (HbA1c) and UDP-glucose during pregnancy, a nucleotide form of glucose important in glycotransferase metabolism. These results suggest that blood glucose rose across gestation in our population and may be a result of insulin resistance that occurs normally during pregnancy. Mannose, which can be produced from glucose, was also elevated during pregnancy in all samples. Mannose is important in fetal development, with 95% of mannose being delivered to the fetus from the mother [29,30]. These results suggest that maternal blood sugars, including glucose and mannose, are elevated across gestation to support the growth and development of the fetus.

Finally, isobutyrate, a metabolite produced from bacteria in the gut, was elevated during pregnancy. This may be due to alterations in gut microbiota during pregnancy, which have been linked to alterations in maternal metabolism and inflammation [31]. In addition, serum creatinine was diminished during pregnancy, which is consistent with increases in the maternal glomerular filtration rate (GFR) due to increased vasodilation and blood flow to the kidneys during pregnancy [32]. Taken together, these results show that there are metabolic alterations in amino acids, lipids, TCA cycle intermediates, and carbohydrates across gestation, regardless of diabetes diagnosis and maternal obesity.

### 3.2. Amino Acid, Carbohydrate, and Lipid Metabolism Are Altered in Women with PGDM during Pregnancy and the Immediate Post-Partum Period

Two pathways involved in amino acid metabolism—alanine/aspartate/glutamate metabolism and glycine/serine/threonine metabolism—were altered in women with PGDM during pregnancy. Isoleucine was significantly higher in early pregnancy in PGDM compared to controls. However, as in controls, circulating isoleucine decreased from early to late pregnancy in PGDM, consistent with increased placental and fetal utilization. This suggests that normal metabolic adaptations to pregnancy still occur in women with PGDM. Isoleucine is an essential, branched-chain amino acid (BCAA) involved in protein synthesis in the muscle. BCAAs are elevated in the blood of non-pregnant, diabetic individuals with insulin resistance and impaired glucose tolerance [33,34]. Yet, few studies have evaluated the impact of PGDM on BCAA alterations in a pregnant population. In rabbit embryos, maternal diabetes during early pregnancy has been shown to significantly elevate BCAAs [35]. Isoleucine was not significantly altered in the immediate post-partum period in women with PGDM, suggesting that physiological metabolic alterations during pregnancy may disrupt changes in BCAA metabolism normally observed in diabetes.

In contrast to isoleucine, we observed significant decreases in glycine, threonine, histidine and glutamine in women with PGDM during pregnancy compared to controls. Diminished circulating glycine has previously been reported as a biomarker for the development of T2D [36,37,38,39], with glycine supplementation improving insulin secretion [40,41]. In addition, threonine has been shown to be significantly diminished in blood from non-pregnant individuals with T2D [42], whereas decreased levels of circulating histidine have been linked to elevated blood glucose, possibly due to hepatic gluconeogenesis [43,44]. While threonine was significant decreased in early pregnancy, threonine rises in women with PGDM by late gestation to levels similar to controls, again suggesting PGDM does not disrupt metabolic adaptations during pregnancy. Finally, glutamine, which is diminished in non-pregnant T2D populations [45], was also significantly lower in PGDM compared to controls. Overall, these results suggest that women with PGDM during pregnancy have similar amino acid metabolic profiles to individuals with diabetes in the general population, especially in early pregnancy and these amino acid metabolic profiles disappear in the immediate post-partum period. However, future studies are needed to determine the mechanisms driving these early pregnancy metabolic differences in women with PGDM and their implications to the placenta and fetus.

Three pathways involved in carbohydrate metabolism—galactose metabolism, glyoxylate/dicarboxylate metabolism, and TCA cycle metabolism—were significantly altered in women with PGDM during pregnancy. Mannose was significantly elevated in PGDM compared to controls, consistent with a previous study of women with diabetes (PGDM and GDM) in late gestation [46]. Mannose is elevated in individuals with diabetes in non-pregnant populations and has been correlated to T2D onset in individuals with insulin resistance [47,48,49]. Succinate and citrate were significantly decreased in PGDM compared to controls across gestation. Previous studies in non-pregnant populations have linked succinate metabolism to endogenous insulin release [50], while decreased succinate was observed in cardiomyocytes cultured in insulin resistant conditions [51]. Decreased circulating succinate and citrate observed in women with PGDM during our study may indicate alterations in TCA cycle energetics, especially in early pregnancy, that disappear in the immediate post-partum period, although the implications this has on the placenta and fetus are unknown.

Finally, one pathway involved in lipid metabolism (synthesis/degradation of ketone bodies) was significantly altered in women with PGDM. While future studies are needed to determine the effects of elevated ketones on the placenta and fetus, our results indicate altered lipolysis and ketogenesis in late gestation in PGDM compared to non-diabetic pregnant women, consistent with impaired glucose control. Lipids were also significantly elevated in PGDM compared to controls following delivery. These latter metabolic alterations were not observed in women with PGDM during pregnancy, suggesting that the elevations in lipids found normally across gestation may mask these diabetes-related metabolic alterations.

### 3.3. Amino Acid, Carbohydrate, and Lipid Metabolism Changes in Women with GDM during Pregnancy

Two pathways involved in amino acid metabolism—glycine/serine/threonine metabolism and alanine/aspartate/glutamate metabolism—were significantly altered in women with GDM during pregnancy. Alterations in glycine/serine/threonine serum metabolism in GDM have previously been reported during the third trimester of pregnancy [52]. Glutamine and threonine are decreased during late gestation [53,54,55], while glycine is significantly decreased during the first and third trimesters with GDM, as well as in non-pregnant cohorts with T2D [36,37,38,39,56,57]. Glutamine showed a significant interaction effect, with decreases in early gestation trending towards significance following a post-hoc analysis (*p* = 0.09). As mentioned above, glutamine decreased in non-pregnant populations with T2D [45]. Therefore, alterations in glutamine, especially in early pregnancy, may serve as a general indicator of diabetes during pregnancy. Future studies in larger populations are needed to determine the implications of these amino acids in the development of GDM.

In addition to amino acid metabolism, two pathways involved in carbohydrate metabolism—glyoxylate/dicarboxylate and butanoate metabolism—were significantly altered in women with GDM. Formate, involved in glyoxylate/dicarboxylate metabolism, was significantly diminished in early pregnancy in women that developed GDM compared to controls. Formate is involved in one-carbon metabolism and is believed to be important in fetal development [58]. While formate has not been previously linked to GDM, decreased urinary formate has been associated with fetal growth restriction, suggesting that depletions may cause poor outcomes at birth [59]. In addition to formate, succinate was also significantly diminished in early pregnancy in women that developed GDM. While this decrease in succinate has not been reported previously in a GDM population, succinate was also significantly decreased in early pregnancy in women with PGDM, suggesting that it may not be specific to GDM development, but also to general glycemic dysregulation during pregnancy. As shown in our results from Section 3.1, formate and succinate did not change across gestation in our population. Therefore, it is interesting that succinate and formate increase in our GDM population throughout gestation to levels similar to controls by late pregnancy, suggesting unique metabolic adaptations that occur due to GDM during pregnancy.

Finally, ketone body degradation/synthesis, associated with altered lipid metabolism, was significantly altered with GDM. Ketone bodies (acetoacetate and 3-hydroxybutyrate), along with glucose, were significantly elevated in women with GDM during late pregnancy compared to controls, suggesting poor glycemic control close to delivery. Furthermore, six lipids were significantly elevated during pregnancy, but not in the immediate post-partum period in our GDM population, suggesting that these alterations are pregnancy-specific. Lipids are elevated in the plasma of women with GDM during pregnancy [53], as well as non-pregnant individuals with T1D or T2D [60,61]. Finally, an NMR resonance generally attributed to *N*-acetyl-glycoproteins was significantly elevated in women with GDM compared to controls. While the exact glycoproteins contributing to this resonance need further investigation, alpha-1-glycoprotein has previously been shown, at least in part, to be a contributor [62]. Alpha-1-glycoprotein is associated with systemic inflammation, which has been linked to GDM development [63]. However, the direct impact of this rise in alpha-1-glycoprotein on GDM development has not been determined.

Three pathways—glyoxylate/dicarboxylate, glycine/serine/threonine, and alanine/aspartate/glutamate metabolism—displayed the same metabolic trends in both women with PGDM and GDM during pregnancy. These results suggest that there are overlapping metabolic changes, even in early gestation, in women that develop GDM to women with diabetes prior to pregnancy. Furthermore, only HbA1c and isoleucine were significantly upregulated in women with PGDM compared to GDM, with HbA1c being significantly elevated at both early and late pregnancy. This suggests that women with PGDM have worsen glycemia control during pregnancy compared to GDM and support the case that there are shared metabolic trends in these women during pregnancy.

Finally, it is worth noting that unlike PGDM which showed metabolic alterations that persisted from pregnancy into the post-partum period, only two compounds were significantly elevated following delivery in GDM compared to controls: HbA1c and mannose. These were also significantly elevated in women with PGDM in the post-partum period, and are not surprising, as glucose dysregulation with GDM is often not fully resolved in the immediate post-partum period. HbA1c is a long-term marker of glucose dysregulation, and elevations in the immediate post-partum period confirm glucose dysregulation in our cohort of women with GDM in late gestation. However, it is interesting that other metabolic alterations seen in women with GDM during pregnancy, even those that overlap with PGDM, disappear immediately following birth. This suggests that while women with GDM during pregnancy still have glucose dysregulation in the immediate post-partum period, other metabolic alterations seen during pregnancy in this population resolve quickly following delivery. Finally, amino acids (including asparagine, leucine, lysine, ornithine, and tyrosine) are significantly upregulated in women with GDM compared to women with PGDM in the post-partum period. While asparagine, ornithine, and tyrosine were significantly decreased in women with PGDM compared to controls, leucine and lysine showed no significant alterations. This further shows that women with PGDM have diminished amino acids following birth, which may be a result of insulin therapy.

Women with GDM during pregnancy are at an increased risk of developing T2D later in life [6]. Our results highlight that there are overlapping alterations in carbohydrate and amino acid metabolism in women with diabetes prior to pregnancy compared to women that develop GDM. However, unlike women with PGDM that had metabolic alterations associated with diabetes in early pregnancy, women with GDM had few metabolites altered in early pregnancy, prior to diabetes diagnosis. This suggests that mechanisms leading to GDM are unique and future studies should focus on how these metabolites and pathways aid in disease progression in women at-risk of developing GDM.

### 3.4. Carbohydrate and Choline Metabolism Are Altered in the Cord Blood of Newborns from Women with PGDM or GDM

Mannose and glucose were significantly decreased in cord blood from newborns of women with diabetes. Mannose was elevated in both PGDM and GDM cord blood compared to controls. Mannose did not change in maternal serum with GDM but was elevated with PGDM compared to controls across both time points. Mannose is an important factor for fetal growth, with the fetus receiving almost all of its mannose from the mother [29,30]. The implications of excess mannose on the fetus are yet to be determined. In addition to mannose, glucose was also significantly elevated in the cord blood of newborns born to women with PGDM, consistent with elevated fetal and newborn glucose in mothers that are diabetic during pregnancy [64,65].

Dimethylglycine, betaine, and glycine (involved in choline metabolism) were significantly altered in women with PGDM or GDM during pregnancy. Dimethylglycine was elevated in PGDM and GDM cord blood compared to controls and is formed during the one-carbon donation of betaine to homocysteine. In addition, dimethylglycine was decreased in women with PGDM during pregnancy, suggesting that this elevation may be maternal in origin. Glycine, a metabolic product of dimethylglycine, was decreased in GDM cord blood compared to controls. While glycine was also decreased in women with PGDM and GDM during pregnancy, no specific interaction effects were observed. Umbilical glycine during late gestation was previously shown to be produced in the fetal–placental unit and not delivered from the maternal circulation [66,67]. Therefore, diminished glycine in the umbilical cord at birth due to maternal diabetes may be from alterations in fetal or placental production. While choline is important in neural development in the fetus [68], the direct implications of changes in its downstream metabolites are not known, and future studies are needed to determine the impact this has on the newborn. Finally, isobutyrate, alanine, and lactate were elevated in PGDM cord blood compared to GDM. Lactate in the cord blood is significantly associated with alanine in human pregnancies [69], with elevations in cord blood lactate being attributed to fetal hypoxia during birth [70].

## 4. Conclusions

In this study, we explored how normal metabolic alterations of pregnancy were disrupted in women with PGDM or GDM compared to non-diabetic controls. We observed elevations in circulating TCA cycle intermediates, carbohydrates, ketones, and lipids, consistent with physiological evidence of increases in maternal basal metabolism, gluconeogenesis, insulin resistance, and lipolysis across gestation. In addition, we found that circulating amino acids were diminished during pregnancy, reflecting elevated placental and fetal uptake.

We observed that women with PGDM in the first trimester of pregnancy had similar metabolic profiles to non-pregnant individuals with T2D in the general population, including elevated isoleucine, but decreased glycine, threonine, histidine, glutamine, and TCA cycle intermediates. However, most of these metabolic profiles were not present in the immediate post-partum period. These insights into metabolic alterations during pregnancy may aid in developing better treatment methods, both during and after pregnancy, in women with diabetes. We also found that women who developed GDM had similar amino acid and TCA cycle metabolic profiles to women with PGDM, but possessed distinct metabolic alterations, including lipid and amino acids levels, especially in early pregnancy. Further research is needed to determine how these metabolic alterations in early pregnancy lead to and/or predict the development of GDM.

Finally, we determined that the cord blood from newborns of mothers with GDM and PGDM had similar metabolic alterations that may not be maternal in origin. Specifically, choline metabolism appeared to be altered in these newborns.

A potential limitation of our study was the low sample size across trimesters for women with PGDM and GDM. Future studies are needed in a larger cohort of individuals to determine trimester-specific differences among these groups. In addition, our cohort was recruited from the high-risk pregnancy clinic at the University of Florida and a majority of subjects, including non-diabetic controls, were obese and nutritional information was not collected in our study. While we believe that our population is large enough to take differences in nutrition into account, future studies should further evaluate how diet may influence metabolic changes during pregnancy. Finally, we were not able to collect umbilical cord serum from all subjects, resulting in a diminished cohort for newborn comparison. However, our study gives preliminary evidence into metabolic alterations that occur during pregnancy longitudinally in both diabetic and non-diabetic obese individuals, and how these normal metabolic changes are altered due to PGDM or GDM. A better understanding of how diabetes during pregnancy alters these normal metabolic transitions can lead to improved diagnostics, better management and more therapeutic options during pregnancy, ultimately leading to improved maternal and neonatal health outcomes.

## 5. Materials and Methods

### 5.1. Sample Collection

Subjects were recruited at the University of Florida Health Shands Hospital High-Risk Maternal-Fetal Medicine Clinic under a protocol approved by the University of Florida Institutional Review Board (UF IRB20150007) and in compliance with guidelines for human research outlined in the Declaration of Helsinki. Subjects gave their written, informed consent for participation prior to enrollment and sample collection. Samples were collected with routine clinical blood draws, and therefore contain a mixed population of fasted and non-fasted samples. Blood was collected in red-top vacutainer tubes (Becton, Dickinson and Company, Franklin Lakes, NJ, USA) and allowed to clot for 1 h at room temperature. Specimens were then centrifuged for 10 min at 3000× *g* at 4 °C, and 400 µL serum was transferred into 1.8 mL cryovials (Thermo Fisher Scientific, Inc, Waltham, MA, USA). Plasma was collected in purple top vacutainer tubes (Becton, Dickinson and Company, Franklin Lakes, NJ, USA) for hemoglobin A1c measurements using a DCA vantage Analyzer (Siemens Healthinners, Malvern, PA, USA). All sera were stored at −80 °C until metabolomic analysis. Maternal blood was collected longitudinally at the first (*n* = 62 control, *n* = 6 PGDM, *n* = 7 GDM), second (*n* = 34 control, *n* = 5 PGDM, *n* = 3 GDM), and third trimester (*n* = 77 control, *n* = 15 PGDM, *n* = 12 GDM), as well as within two days post-partum (*n* = 57 control, *n* = 12 PGDM, *n* = 12 GDM). Umbilical cord blood was collected from the clamped umbilical cord immediately following delivery of the placenta (*n* = 22 control, *n* = 6 PGDM, *n* = 7 GDM). Women with pre-gestational diabetes received insulin, while those diagnosed with GDM during pregnancy did not receive medication for blood sugar control, per standard of care. All maternal demographic information is listed in Supplementary Table S10 for maternal serum and Supplementary Table S11 for umbilical cord serum cohorts. A Student’s t-test was used to determine significant differences between PGDM and controls or GDM and controls for maternal age, BMI, HbA1c, and gestational age at delivery (*p* < 0.05). HbA1c measurements were missing for the following maternal specimens and were therefore excluded from analysis: first trimester (*n* = 4 control), second trimester (*n* = 1 control), third trimester (*n* = 4 control, *n* = 1 PGDM), and post-partum (*n* = 5 control, *n* = 1 PGDM, *n* = 1 GDM).

### 5.2. Metabolomic Analysis

Proton nuclear magnetic resonance (^1^ H-NMR) was used to quantify metabolites in maternal and umbilical cord serum. Samples were thawed at 4 °C and centrifuged at 46,000× *g* for 40 min at 4 °C. Next, specimens were transferred into a 5 mm SampleJet NMR tube (Bruker Biospin, Billerica, MA, USA) with a SamplePro Tube robotic system (Bruker Biospin, Billerica, MA, USA). Samples were prepared as previously described [71]. Briefly, 300 μL 100 mM sodium phosphate buffer at pH 7 with 0.33 mM DSS-D6 was added to NMR tubes, then 300 μL of serum was added and mixed. Samples were analyzed on a Bruker Avance III-HD 600 MHz NMR spectrometer equipped with a 5 mm cryoprobe and Bruker SampleJet cooled to 5.6 °C at the University of Georgia Complex Carbohydrate Research Center. Metabolite measurements were acquired using a one-dimensional experiment with T2 filter using a PROJECT pulse sequence with water pre-saturation [72], while lipid measurements were obtained using a one-dimensional diffusion-edited experiment with water pre-saturation (ledbpgppr2s1d).

Two-dimensional (2D) NMR spectroscopy was used to aid in annotation of metabolites detected in 1D NMR data of serum specimens and was not used for statistical analyses. Data were acquired using ^1^H-^13^C heteronuclear single quantum correlation (HSQC) and ^1^H-^13^C HSQC–TOCSY (HSQC–total correlation spectroscopy) experiments. A total of 52 metabolites were identified in maternal and umbilical cord serum using COLMARm [73], and assigned a confidence level ranging from 1 to 5, as previously described [74]. The spectra were processed using Bruker Topspin 3.6 software and in-house MATLAB scripts (https://github.com/artedison/Edison_Lab_Shared_Metabolomics_UGA). The exact spectral areas for integration and confidence values for each metabolite included in statistical analysis are listed in Supplementary Table S1. Detailed experimental NMR methods, as well as all raw and processed data are available on the Metabolomics Workbench (http://www.metabolomicsworkbench.org/).

### 5.3. Statistical Analysis

Multi- and univariate statistics were performed on metabolites identified in maternal and umbilical cord serum using 1D NMR data after PQN normalization. Multivariate statistical analysis was performed using Metaboanalyst 4.0 [75] and univariate analyses were performed using R and MATLAB. Individuals with more than one sample per time point were averaged prior to multivariate and univariate analysis. To determine significant trimester-specific effects throughout pregnancy in maternal serum, differences in metabolites were analyzed using a linear mixed-effects model in R [76], taking into account a random effect (subject), random error (within subjects), two fixed effects (trimester and PGDM/GDM diagnosis), and a covariate (BMI). Trimester effects were considered significant with a false discovery rate (FDR)-corrected *p* < 0.10. For significant metabolites, a Tukey–Kramer post-hoc test was conducted to determine significant alterations over each trimester (*p* < 0.05).

Differences in metabolites due to PGDM or GDM during pregnancy were analyzed using a linear mixed-effects model in R [76], taking into account a random effect (subject), random error (within subjects), two fixed effects (time point and PGDM or GDM diagnosis), a covariate (BMI), and an interaction effect (time point*PGDM or GDM). This model was run three times: once with PGDM and control subjects, once with GDM and control subjects, and once with PGDM and GDM subjects. Due to the low availability of samples in the second trimester, the following time points were used in each analysis to maximize the amount of diabetic samples: (1) PGDM vs. controls: early gestation (<20 weeks) and late gestation (>30 weeks), and (2) GDM vs. controls: early gestation (<18 weeks) and late gestation (>30 weeks). In addition, due to low sample size for diabetic subjects, individual metabolites were considered significantly changed if they had a raw *p* < 0.05 for diabetes (PGDM or GDM) and interaction effects (time point*PGDM or GDM). For metabolites with significant interaction effects between trimester and diabetes, a Tukey–Kramer post-hoc test was conducted to determine significant alterations over each trimester (*p* < 0.05). Pathway analysis was conducted using Metaboanalyst with significant metabolites from each linear mixed-effects model as previously described [75,77]. Pathways not related to human metabolism or containing D-amino acids were removed from the results. Pathways with a raw *p*-value < 0.05 were considered significant.

To determine the effect of diabetes in the immediate post-partum period, we determined metabolite concentrations in maternal serum within two days following delivery. Differences in metabolites for PGDM compared to controls and GDM compared to controls were analyzed using a linear regression model taking into account a covariate (BMI). Individual metabolites were considered significantly changed if they had a raw *p* < 0.05 for diabetes (PGDM or GDM).

A Student’s t-test was used to determine metabolites altered in serum from the umbilical cord in women with diabetes compared to controls. Two comparisons were made: (1) women with PGDM compared to controls; and (2) women with GDM compared to controls.

## Figures and Tables

**Table 1:** Significantly Altered Metabolites over Gestation, Regardless of Diabetes Diagnosis.

Metabolite	*p*-Value	FDR	Change during Pregnancy (±)
HbA1c ^1^	4.22 × 10^−3^	5.33 × 10^−3^	+
*Amino Acids*			
Alanine	7.34 × 10^−4^	1.01 × 10^−3^	−
Arginine	1.72 × 10^−9^	4.29 × 10^−9^	−
BCAA ^2^	4.44 × 10^−15^	1.68 × 10^−14^	−
Dimethylglycine	1.55 × 10^−4^	2.29 × 10^−4^	−
Glutamate	1.41 × 10^−5^	2.26 × 10^−5^	+
Glutamine	3.42 × 10^−18^	1.51 × 10^−17^	−
Glycine	7.43 × 10^−4^	1.01 × 10^−3^	−
Histidine	8.38 × 10^−5^	1.27 × 10^−4^	−
Isoleucine	2.17 × 10^−21^	1.44 × 10^−20^	−
Leucine	5.21 × 10^−5^	8.12 × 10^−5^	−
Lysine	1.86 × 10^−19^	9.86 × 10^−19^	−
Ornithine	6.60 × 10^−15^	2.33 × 10^−14^	−
Phenylalanine	4.12 × 10^−8^	7.53 × 10^−8^	+
Proline	4.71 × 10^−11^	1.39 × 10^−10^	−
Serine	1.37 × 10^−5^	2.26 × 10^−5^	−
Threonine	1.46 × 10^−3^	1.89 × 10^−3^	+
Tyrosine	4.11 × 10^−8^	7.53 × 10^−8^	−
Valine	8.08 × 10^−25^	1.07 × 10^−23^	−
*TCA Cycle*			
Citrate	3.34 × 10^−9^	7.38 × 10^−9^	+
Lactate	9.04 × 10^−9^	1.84 × 10^−8^	+
Pyruvate	8.08 × 10^−11^	2.25 × 10^−10^	+
*Carbohydrates*			
Mannose	1.46 × 10^−3^	1.89 × 10^−3^	+
UDP-Glucose ^3^	1.52 × 10^−8^	2.98 × 10^−8^	+
*Ketones*			
Acetoacetate	5.61 × 10^−4^	8.03 × 10^−4^	+
Acetone	1.87 × 10^−9^	4.31 × 10^−9^	+
3-Hydroxybutyrate	3.50 × 10^−6^	5.98 × 10^−6^	+
*Lipids*			
CH3 fatty acids	9.37 × 10^−34^	4.97 × 10^−32^	+
(-CH2)n fatty acids	1.27 × 10^−24^	1.35 × 10^−23^	+
Beta-CH2 fatty acids	3.92 × 10^−23^	3.46 × 10^−22^	+
CH_2_CH=C fatty acids	1.30 × 10^−26^	2.30 × 10^−25^	+
Alpha-CH2 fatty acids	7.18 × 10^−19^	3.46 × 10^−18^	+
C=CCH_2_C=C fatty acids	8.87 × 10^−23^	6.72 × 10^−22^	+
Glyceryl C1,3H	4.08 × 10^−12^	1.35 × 10^−11^	+
Glyceryl C2H	4.75 × 10^−20^	2.80 × 10^−19^	+
CH=CH fatty acids	8.32 × 10^−28^	2.20 × 10^−26^	+
*Other*			
Betaine	1.78 × 10^−9^	4.29 × 10^−9^	−
Glycolipids (GPC/PC) ^3,4^	1.96 × 10^−10^	5.19 × 10^−10^	−
*N*-acetyl-glycoproteins	2.28 × 10^−6^	4.03 × 10^−6^	+
Isobutyrate	1.11 × 10^−11^	3.46 × 10^−11^	+
Myoinositol	6.25 × 10^−3^	7.70 × 10^−3^	−
Creatinine	3.21 × 10^−15^	1.31 × 10^−14^	−
Dimethylsulfone	8.56 × 10^−9^	1.81 × 10^−8^	−

^1^ HbA1c: hemoglobin A1c; ^2^ BCAA: branched-chain amino acids; ^3^ UDP: uridine diphosphate; ^3^ GPC: glycerophosphocholine; ^4^ PC: phosphocholine.

**Table 2:** Metabolites Significantly Altered in Maternal Serum in PGDM or GDM during Pregnancy.

Metabolite	*p*-Value	FDR
**PGDM ^1^ vs. Controls**		
*Increased PGDM*		
HbA1c ^2^	4.81 × 10^−21^	2.55 × 10^−19^
Glucose	1.64 × 10^−3^	0.04
Isoleucine	7.47 × 10^−3^	0.08
BCAA ^3^	0.04	0.15
Mannose	0.04	0.15
*Decreased PGDM*		
Glycerol	4.62 × 10^−3^	0.08
Creatinine	6.24 × 10^−3^	0.08
Threonine	0.01	0.10
Succinate	0.01	0.11
Glutamine	0.02	0.12
Glycine	0.03	0.15
Myoinositol	0.03	0.15
Histidine	0.03	0.15
Citrate	0.03	0.15
Dimethylglycine	0.04	0.15
**GDM ^4^ vs. Controls**		
*Increased GDM*		
Glyceryl C2H	0.01	0.16
Glyceryl C1,3H	0.02	0.16
Alpha-CH2 fatty acids	0.03	0.16
*N*-acetyl-glycoproteins	0.03	0.16
CH_2_CH=C fatty acids	0.04	0.16
(-CH2)n fatty acids	0.04	0.16
CH=CH fatty acids	0.04	0.16
*Decreased GDM*		
Succinate	0.01	0.16
Formate	0.02	0.16
Serine	0.02	0.16
Threonine	0.03	0.16
Isobutyrate	0.03	0.16
Acetate	0.03	0.16
Glutamine	0.05	0.16
Glycine	0.05	0.16
Creatinine	0.05	0.16

^1^ PGDM: pre-gestational diabetes; ^2^ HbA1c: hemoglobin A1c; ^3^ BCAA: branched-chain amino acids; ^4^ GDM: gestational diabetes mellitus.

**Table 3:** Metabolites with Significant Interaction Effects in Maternal Serum in PGDM or GDM throughout Pregnancy.

Metabolite	*p*-Value	<20 Weeks	>30 Weeks
**PGDM ^1^ vs. Controls**			
HbA1c ^2^	1.23 × 10^−4^	PGDM > Ctrl	PGDM > Ctrl
Threonine	0.01	Ctrl > PGDM	-
Succinate	0.02	Ctrl > PGDM	-
Isoleucine	0.03	PGDM > Ctrl	-
Acetate	0.03	-	PGDM > Ctrl
Acetone	0.04	-	PGDM > Ctrl
**GDM ^3^ vs. Controls**		**<18 Weeks**	**>30 Weeks**
Acetoacetate	2.32 × 10^−3^	-	GDM > Ctrl
Succinate	6.44 × 10^−3^	Ctrl > GDM	-
Acetate	0.01	-	-
Formate	0.02	Ctrl > GDM	-
3-Hydroxybutyrate	0.02	-	GDM > Ctrl
Glucose	0.03	-	GDM > Ctrl
Glutamine	0.04	-	-

^1^ PGDM: pre-gestational diabetes; ^2^ HbA1c: hemoglobin A1c; ^3^ GDM: gestational diabetes mellitus.

**Table 4:** Pathways Significantly Altered in Maternal Serum in PGDM or GDM throughout Pregnancy.

Pathway	Class	Significant Metabolites		*p*-Value	FDR
**PGDM ^1^ vs. Controls**		*Increased*	*Decreased*		
Galactose metabolism	Carbohydrate Metabolism	Glucose, Mannose	Glycerol, Myo-inositol	8.77 × 10^−5^	3.68 × 10^−3^
Glyoxylate and dicarboxylate metabolism	Carbohydrate Metabolism	Acetate	Citrate, Glycine, Glutamine	1.75 × 10^−4^	4.89 × 10^−3^
Alanine, aspartate and glutamate metabolism	Amino Acid Metabolism	-	Glutamine, Citrate, Succinate	2.08 × 10^−3^	0.04
Glycine, serine and threonine metabolism	Amino Acid Metabolism	-	Dimethylglycine, Glycine, Threonine	3.36 × 10^−3^	0.05
Citrate cycle (TCA cycle)	Carbohydrate Metabolism	-	Succinate, Citrate	0.02	0.18
**GDM ^2^ vs. Controls**		*Increased*	*Decreased*		
Glyoxylate and dicarboxylate metabolism	Carbohydrate Metabolism	-	Serine, Glycine, Acetate, Formate, Glutamine	1.94 × 10^−6^	1.63 × 10^−4^
Butanoate metabolism	Carbohydrate Metabolism	3-Hydroxybutyrate, Acetoacetate	Succinate	1.53 × 10^−4^	6.44 × 10^−3^
Synthesis and degradation of ketone bodies	Lipid Metabolism	3-Hydroxybutyrate, Acetoacetate	-	5.43 × 10^−4^	0.01
Glycine, serine and threonine metabolism	Amino Acid Metabolism	-	Glycine, Serine, Threonine	1.70 × 10^−3^	0.03
Alanine, aspartate and glutamate metabolism	Amino Acid Metabolism	-	Glutamine, Succinate	0.02	0.22

^1^ PGDM: pre-gestational diabetes; ^2^ GDM: gestational diabetes mellitus.

**Table 5:** Metabolites Significantly Altered in Maternal Serum in PGDM or GDM during the Immediate Post-Partum Period.

Metabolite	*p*-Value	FDR	Trend
**PGDM ^1^ vs. Controls**			
HbA1c ^2^	4.28 × 10^−12^	2.27 × 10^−10^	PGDM > ctrl
Mannose	3.87 × 10^−4^	0.01	PGDM > ctrl
Glucose	1.54 × 10^−3^	0.03	PGDM > ctrl
*Amino acids*			
Histidine	4.50 × 10^−3^	0.06	Ctrl > PGDM
Tyrosine	0.01	0.10	Ctrl > PGDM
Asparagine	0.03	0.12	Ctrl > PGDM
Glutamine	0.03	0.12	Ctrl > PGDM
Ornithine	0.04	0.12	Ctrl > PGDM
Methionine	0.05	0.12	Ctrl > PGDM
*Ketones*			
Acetone	0.01	0.11	PGDM > ctrl
Acetoacetate	0.04	0.12	PGDM > ctrl
3-Hydroxybutyrate	0.05	0.12	PGDM > ctrl
*Lipids*			
(-CH2)n fatty acids	0.02	0.12	PGDM > ctrl
Alpha-CH2 fatty acids	0.03	0.12	PGDM > ctrl
Glyceryl C1,3H	0.03	0.12	PGDM > ctrl
Beta-CH2 fatty acids	0.04	0.12	PGDM > ctrl
Glyceryl C2H	0.04	0.12	PGDM > ctrl
CH_2_CH=C fatty acids	0.04	0.12	PGDM > ctrl
*N*-acetyl-glycoproteins	0.04	0.12	PGDM > ctrl
*Other*			
Betaine	0.05	0.12	PGDM > ctrl
Creatinine	9.95 × 10^−3^	0.10	Ctrl > PGDM
**GDM ^3^ vs. Controls**			
HbA1c ^2^	3.03 × 10^−3^	0.16	GDM > Ctrl
Mannose	0.02	0.61	GDM > Ctrl

^1^ PGDM: pre-gestational diabetes; ^2^ HbA1c: hemoglobin A1c; ^3^ GDM: gestational diabetes mellitus.

**Table 6:** Pathways Significantly Altered in Maternal Serum in PGDM during the Immediate Post-Partum Period.

Pathway	Class	Significant Metabolites		*p*-Value	FDR
**PGDM ^1^ vs. Controls**		*Increased*	*Decreased*		
Synthesis and degradation of ketone bodies	Lipid Metabolism	3-Hydroxybutyrate, Acetoacetate	-	4.53 × 10^−4^	0.02
Arginine biosynthesis	Amino Acid Metabolism	-	Ornithine, Glutamine	3.98 × 10^−3^	0.10
Butanoate metabolism	Carbohydrate Metabolism	3-Hydroxybutyrate, Acetoacetate	-	4.57 × 10^−3^	0.10
Galactose metabolism	Carbohydrate Metabolism	Glucose, Mannose	-	0.01	0.19
Alanine, aspartate and glutamate metabolism	Amino Acid Metabolism	-	Asparagine, Glutamine	0.02	0.19
Phenylalanine, tyrosine and tryptophan biosynthesis	Amino Acid Metabolism	-	Tyrosine	0.03	0.30
Tyrosine metabolism	Amino Acid Metabolism	Acetoacetate	Tyrosine	0.03	0.32

^1^ PGDM: pre-gestational diabetes.

**Table 7:** Metabolites Significantly Altered in Cord Blood in PGDM or GDM.

Metabolite	*p*-Value	FDR	Mean Control (SE)	Mean Diabetes (SE)	FC (Diabetes/Control)
**PGDM ^1^ (*n* = 6) vs. Controls (*n* = 22)**					
Mannose	0.02	0.53	0.06 (0.003)	0.08 (0.009)	0.34
Glucose	0.02	0.53	1.86 (0.083)	2.33 (0.214)	0.33
Dimethylglycine	0.05	0.59	0.02 (0.001)	0.02 (0.003)	0.31
**GDM ^2^ (*n* = 7) vs. Controls (*n* = 22)**					
Mannose	3.92 × 10^−4^	0.02	0.06 (0.003)	0.09 (0.005)	0.44
Dimethylglycine	0.02	0.40	0.02 (0.001)	0.02 (0.003)	0.39
Glycine	0.03	0.40	0.87 (0.034)	0.72 (0.036)	−0.27
Betaine	0.03	0.40	0.26 (0.005)	0.28 (0.013)	0.14

^1^ PGDM: pre-gestational diabetes; ^2^ GDM: gestational diabetes mellitus.

## Supplementary Materials

The following are available online at https://www.mdpi.com/2218-1989/10/9/350/s1. The following are in the attached Supplementary Material document: Tables S1–S11.
